# Tissue Engineering in Gene and Cell Therapies for Neurological Disorders

**DOI:** 10.1155/2016/8738504

**Published:** 2016-04-20

**Authors:** Jun Liu, William Z. Suo, Xing-Mei Zhang, Chen Zhang, Gongxiong Wu

**Affiliations:** ^1^Department of Neurology, Sun Yat-sen Memorial Hospital, Sun Yat-sen University, Guangzhou, Guangdong 510120, China; ^2^Laboratory for Alzheimer's Disease and Aging Research, Veterans Affairs Medical Center, Kansas City, MO 64128, USA; ^3^Department of Neurology, University of Kansas Medical College, Kansas City, MO 64128, USA; ^4^Immunology-Immunotherapy, Department of Clinical Neuroscience, Karolinska Institutet, 171 76 Stockholm, Sweden; ^5^State Key Laboratory of Membrane Biology, School of Life Sciences, PKU-IDG/McGovern Institute for Brain Research, Peking University, Beijing 100871, China; ^6^Laboratory for Translational Research, Harvard Medical School, Cambridge, MA 02215, USA

Neurological diseases such as stroke, encephalitis, neurologic tumors, and neurodegenerative diseases are far from alien to us. These diseases are refractory, harmful, and sometimes devastating. The pathogenesis of some nervous system diseases remains elusive, especially the neurodegenerative diseases such as AD and PD. Many therapeutic strategies have been developed to potentially intervene in these progressive neurodegenerative events and minimize damage to the CNS. Despite the promising results that these researches have shown in in vivo and in vitro studies, challenges still remain, particularly when it comes to clinical translation. The causes are various, which may include biologic and physiologic differences between the preclinical models and the human condition and discrepancies in dosing and the timing of drug therapy. The last few years have witnessed an explosion in the use of genes and cells as biomedicines. Gene and cell therapies are researches with the goals of repairing the direct cause of genetic diseases in the DNA or cellular population, respectively. In many diseases, gene and cell therapies are combined in the development of promising therapies, which have helped provide reagents, concepts, and techniques that are elucidating the finer points of gene regulation, stem cell lineage, regenerative capacity, and remodeling. Additionally, increasing studies of novel drug delivery systems bring more considerable future on gene and cell therapies. This special issue mainly focuses on the latest ideas, developments, and applications in the field of gene and cell therapies for neurological disorders, most notably development of highly efficient gene expression and delivery system including viral and nonviral gene vectors for tissue repair in the nervous system; exploration and evaluation of the efficiency of new ideal routes of administration of gene and cells into brain; evaluation of appropriate biomaterials in terms of biocompatibility and biodegradability, biomechanical competency, and functionalization with instructive cues to guide neuroregeneration; tissue engineering that integrates cell therapies and scaffolding technologies to replace damaged neurons and to reestablish the coaxial connection of neuronal circuits for functional recovery; assessment of the safety and toxic side effects with gene and cell therapies; latest technologies of noninvasive in vivo imaging to track the transplanted gene or cells; new insights into the pathogenesis of neurological disorders by using cellular or animal models.

In the accepted papers, researches of pathogenesis of some diseases, such as A*β*-induced autophagy in AD and ischemia-reperfusion injury induced apoptosis in stroke, were discussed. Studying specific aptamer for GP73 through systematic evolution of ligands by exponential enrichment and identifying its application in detecting hepatocellular carcinoma might provide a novel molecular probe for cell therapy. In vivo targeted MR imaging of endogenous neural stem cells (NSCs) in adult mouse brain by using certain nanoparticles offered a promising molecular probe in NSCs research. Moreover, the role of Nogo-A in PD and NTRK1 in NSCs differentiation was preliminarily explored. These findings above provided new insights into the pathogenesis of certain diseases and also represented part of the latest ideas, developments, and applications in the field of gene and cell therapies for neurological disorders.

*Jun Liu* *William Z. Suo* *Xing-Mei Zhang* *Chen Zhang* *Gongxiong Wu*

